# Embracing change: impermanence acceptance mediates differences in death processing between long-term ayahuasca users and non-users

**DOI:** 10.1007/s00213-025-06792-0

**Published:** 2025-04-23

**Authors:** Jonathan David, Aviva Berkovich-Ohana, Yair Dor-Ziderman

**Affiliations:** 1 Edmond J. Safra Brain Research Center, University of Haifa, Haifa, Israel; 2 Integrated Brain and Behavior Research Center (IBBRC), University of Haifa, Haifa, Israel; 3 School of Therapy, Counseling, and Human Development, Faculty of Education, University of Haifa, Haifa, Israel

**Keywords:** Ayahuasca, Psychedelics, Death acceptance, Death anxiety, Fear of death, Impermanence, Ego dissolution

## Abstract

**Rationale:**

The human psyche's interaction with death fundamentally shapes cognition, emotions, and behavior in both individuals and society. Death-related psychological phenomena have been shown to be influenced by psychedelic interventions. However, the literature lacks a comprehensive assessment of death-related processes in non-clinical settings, the mechanisms underlying long-term changes, and particularly the effects of ayahuasca on these dimensions.

**Objectives:**

This cross-sectional study investigates death processing, potential mechanisms of change, and their predictors in ayahuasca veterans (N = 54) compared to non-users (N = 53).

**Methods:**

A battery of questionnaires and behavioral assessments were used to evaluate different aspects of death processing in both ayahuasca veterans and non-users. These assessments measured death fear and anxiety, death-acceptance, death-avoidant behaviors, and the accessibility of death-related thoughts. Mediators tested included personality traits, beliefs about the afterlife, trait mindfulness, and the concept of impermanence.

**Results:**

The findings demonstrated lower levels of death anxiety, avoidant behavior, and fear of death, as well as greater death acceptance in ayahuasca veterans. Mediation analyses revealed that group differences were not due to demographics, personality, trait mindfulness, ontological beliefs, or impermanence awareness, but rather to impermanence acceptance. Finally, within the ayahuasca group, lifetime ego dissolution experiences predicted the degree of impermanence acceptance.

**Conclusions:**

These findings reveal significant, multi-dimensional differences in death processing between ayahuasca and non-psychedelic users. Impermanence acceptance emerged as the key mechanism of change. Additionally, the results highlight the role of acute ayahuasca experiences in producing lasting effects. Future interventions may focus on promoting impermanence acceptance as a strategy for managing existential fear.

**Supplementary Information:**

The online version contains supplementary material available at 10.1007/s00213-025-06792-0.

* “..through ceremonies where you just have to endure through the night and you go through everything, so especially there, in the integration into daily life you come to understand that the morning will come. This means it will pass. This anger, this unpleasant interaction... it will pass, like everything else, like a mosquito buzzing during meditation, like when during a ceremony when we are at pit bottom but later we will dance with joy. This means you understand that everything is impermanent. You can get there through other ways. But certainly,… she [ayahuasca] opens the door for you to the understanding that everything is temporary, that everything is impermanent.”*

participant 543

## Introduction

Death is an undeniable universal truth that profoundly affects human existence, shaping both individual psychology and societal structures. It permeates both physical and psychological well-being, compelling individuals to seek meaning and develop coping strategies in response to the existential challenge it presents. Throughout history and across diverse cultures, humanity has developed numerous and diverse approaches to dealing with the existential terror posed by mortality (Arndt et al. 2005; Kastenbaum and Moreman 2018). Both Amazonian indigenous cultures and contemporary scientific studies emphasize a profound association between ayahuasca, a potent psychedelic brew containing N,N-Dimethyltryptamine (DMT) and harmala alkaloids, and the theme of death. In Quechua languages (from where the term ayahuasca originates), ayahuasca means the ‘dead-liana’ (Brabec de Mori 2011) or the ‘vine of death’ (Shanon 2002). The noted anthropologist Reichel-Dolmatoff (1971) observed that, for some indigenous Amerindians, "to take Yage [ayahuasca, was] to die" (Shanon 2002), and there is archeological evidence suggesting the Inca used ayahuasca plants in ceremonies associated with death (Socha et al. 2022). Drawing from approximately 2,500 descriptions of ayahuasca experiences, Shanon (2002) concluded that themes related to death are among the most prominent in ayahuasca visions. These themes encompass a diverse range of phenomena, including the subjective sense of personal death, death-rebirth and near-death like experiences, ‘encounters’ with deceased individuals, visions or hallucinations of dead bodies and death angels, and themes related to the afterlife. Recent quantitative studies corroborate Shanon's phenomenological findings regarding the high prevalence of death-related themes in ayahuasca experiences. A recent cross-sectional study from our lab (David et al. 2023) indicates that personal-death experiences during ayahuasca ceremonies are prevalent, with around 50% of general ayahuasca users and more than 65% of veterans reporting having previously experienced these phenomena. Another study by González et al. (2019), testing the potential use of ayahuasca in grief therapy, found that over 50% of the participants reported experiencing direct ‘contact’ with a deceased individual during ayahuasca interventions.

Given ayahuasca's deep connection to the theme of death, one might expect that its effect on human conceptions, emotions, cognition and behaviour around mortality would be empirically studied. However, research in this area remains limited. These include a few cross-sectional online survey studies on death-related themes which include ayahuasca participants as part of their psychedelic database, but do not report explicitly on them (Moreton et al. 2025; Moreton et al. 2023b; Moreton et al. 2023a). To our knowledge, the only studies providing direct evidence are two internet-based surveys examining death-related aspects among psychedelic users, including subgroups of ayahuasca users (Griffiths et al. 2019; Sweeney et al. 2022). In Griffiths et al. (2019), participants were questioned about perceived changes in fear of death following a "god encounter" subjective experience, with 73% of ayahuasca users (n = 435) reporting a decrease in fear of death post-experience. Sweeney et al. (2022) administered the Revised Death Attitude Profile (DAP-R) scale (Wong et al. 1994) to assess participants' attitudes toward death before and after psychedelic experiences. Ayahuasca users reported a reduction in fear of death and death anxiety alongside decreases in death avoidance and positive shifts in curiosity and interest towards death (Sweeney et al. 2022). A full review of the effects of psychedelic substances other than ayahuasca on death processing falls beyond the purview of this article. However, a number of studies, mostly on psilocybin and LSD, have demonstrated decreases in death anxiety and increases in death acceptance among users or after psychedelic use (Davis et al. 2020; Gasser et al. 2015; Griffiths et al. 2011, 2016, 2018, 2019; Moreton et al. 2025; Moreton et al. 2023a; Moreton et al. 2023b; Sweeney et al. 2022; Yaden et al. 2017).

Observing this growing body of literature, it's important to recognize its limitations. Firstly, all the mentioned studies rely exclusively on self-report measures, which are inherently limited to what is consciously accessible. This issue becomes crucial in the context of studying human death-related aspects, where unconscious denial and defensive processes are prevalent (Dor-Ziderman et al. 2019; Pyszczynski et al. 2015). Secondly, the studies primarily revolve around singular metrics, rather than exploring the broader construct of human relationship with death, which we term here 'death processing'. This term encapsulates a spectrum of affective, cognitive, behavioral, and conceptual levels associated with how death is processed. Thirdly, and specifically related to the mentioned ayahuasca studies, these lacked control groups and were all conducted through online platforms, raising concerns about potential biases (Buchanan 2018). Thus, our current study aims (Aim 1) to address these gaps and limitations by investigating death processing in veteran ayahuasca users compared to non-users controls, utilizing a comprehensive battery of questionnaires and behavioral measures designed to evaluate death processing, with both explicit and implicit indicators (see Table 1 and Measures section).

**Table 1:** The table provides an overview of the death processing measures utilized in the study, outlining their type (self-report or behavioral), the constructs they measure, the domain they target (affective/cognitive/behavior), their novelty in the psychedelic literature, and our related hypotheses (upward/downward arrows indicate hypothesized increase/decrease for ayahuasca veterans compared to controls)

Death processing measure	Type	Construct	Domain	Novelty	Hypotheses
Death Anxiety Scale (DAS)	Self-report	Death anxiety	Affect	Previously used (David et al. 2023; Ross et al. 2016)	↓
Death behaviors Questionnaire (DBQ)	Self-report	Death-avoidant behavior	Behavior	Novel	↓
Life Attitude Profile-Revised (LAP-RD)	Self-report	Death acceptance	Attitudes	Previously used (Griffiths et al. 2016, 2018)	↑
Fear of Personal Death Scale percentage (FPDS_P)	Self-report	Fear of death	Affect	Novel	↓
Fear of Personal Death Scale reaction times (FPDS_RT)	Behavioral	Emotional load	Affect	Novel	↓
Death Thoughts Accessibility (DTA)	Behavioral	Death-thoughts suppression	Cognition	Novel	↓

The study also aims (Aim 2) to address an open question in the literature regarding the mechanisms underlying the effects of psychedelics on death processing. In a recent opinion paper, Letheby (2024) made the case, based on mainly qualitative and anecdotal data, but also some quantitative evidence (Moreton et al. 2025), that psychedelics substantially and durably reduce the fear of death by promoting non-physicalist metaphysical beliefs. Indeed, clinical trials have demonstrated that psychedelics modify human ontological representations of death by increasing beliefs in after-death continuation (Griffiths et al. 2011, 2018). Additionally, recent large-scale surveys also demonstrate that psychedelic experiences are associated with increases in non-physicalist beliefs such as reincarnation, communication with the dead, and existence of consciousness after death (Nayak et al. 2023), and shifts away from materialist views towards panpsychism and fatalism (Timmermann et al. 2021). Beyond psychedelic populations, beliefs in after-death continuation have been associated with reduced death anxiety (Harding et al. 2005; Reece et al. 2023; Taghiabadi et al. 2017) and higher death acceptance (Falkenhain and Handal 2003). Finally, terror management theory (TMT) holds that beliefs in after-life continuation, which they term literal immortality, serve as a buffer against basic human death anxiety (Dechesne et al. 2003; Fan et al. 2023; Piwowarski et al. 2011; Reece et al. 2023). These data make a case for altered ontological beliefs as the mechanism-of-action underlying changes in death processing as previously proposed (Letheby 2024; Moreton et al. 2020).

Another candidate mediator is a person’s personality, which has been shown to differ for psychedelic populations compared to non-users (Anglin et al. 1986; Barbosa et al. 2016; Bouso et al. 2012, 2015; Kavenská and Simonová, 2015; Schneider et al. 2015). Recent research goes further in showing that psychedelics induce enduring changes to certain personality traits, in particular significant increases in openness to experience (Erritzoe et al. 2018; MacLean et al. 2011; Madsen et al. 2020; Netzband et al. 2020), and reductions in neuroticism personality traits (Erritzoe et al. 2018; Netzband et al. 2020; Weiss et al. 2021). Additionally, though less consistently, there have been reported increases in absorption and conscientiousness following psychedelic use (Barrett et al. 2020). On the other hand, personality traits play a role in determining how individuals' approach death. Research has identified relationships between degree of death anxiety and personality traits such as neuroticism (Frazier and Foss-Goodman 1989; Galton et al. 2022; Khizer 2021; Mavrogiorgou et al. 2023) and extraversion (Khizer 2021). Additionally, individuals characterized by higher openness have demonstrated a tendency to exhibit less defensive reactions when confronted with thoughts of mortality (Boyd et al. 2017). Overall, these findings imply that the impact of psychedelics on long-term death processing may be mediated by either different population baseline personality traits or psychedelic-induced alterations to these traits.

We also tested whether trait mindfulness mediated group differences in death processing. Cross sectional studies have associated psychedelic use to higher trait mindfulness (Sampedro et al. 2017), and a number of studies have reported increases in mindfulness following psychedelic interventions (Madsen et al. 2020; Smigielski et al. 2019a, b; Søndergaard et al. 2022; Uthaug et al. 2019), and ayahuasca usage in particular (Domínguez-Clavé et al. 2019; Murphy-Beiner and Soar 2020; Sampedro et al. 2017; Soler et al. 2016, 2018; Uthaug et al. 2018). More generally, mindfulness meditation training has been shown to decrease explicit fear of death and dying (Anālayo et al. 2022), as well as unconscious thought suppression and defensiveness (Park and Pyszczynski 2019). Finally, a large TMT study on trait mindfulness established through seven experiments that it reduced defensive responses as well as thought suppression to mortality salience (Niemiec et al. 2010). Together, these studies make a case for mindfulness as a possible mediator for group differences in death processing.

Finally, we present a novel candidate mediator with deep contemplative cross-cultural roots, namely, the construct of impermanence. The concept of impermanence refers to the uncertain and transient nature of what we tend to regard as a stable reality. A famous saying attributed to Heraclitus states that “No man ever steps in the same river twice, for it's not the same river and he's not the same man” (Weisel 2010). Ilya Prigogine, the Nobel Laureate for nonequilibrium thermodynamics, argued for the notion of inherent unpredictability as one of nature’s fundamental features (Prigogine 1997). The Buddhist contemplative tradition perhaps takes impermanence (in Pali, *aniccā*) even further, holding it as one of the three basic facts of existence (Thera 1981), and developing methods for cultivating awareness and acceptance of it across all Buddhist schools (Gokhale 2021). Inspired by the Buddhist tradition, Fernández-Campos et al. (2021) recently developed a self-report tool for the measurement of impermanence, where they distinguish between a cognitive and affective component. The cognitive component is the awareness that all phenomena, including thoughts, emotions, and all animate and inanimate objects are subject to change and dissolution; the affective component involves a sense of openness and ease towards the transient nature of all phenomena – the embracing of change. The resulting 13-item, dual-subscale questionnaire showed good model fit, reliability, and convergent validity with other constructs, including death acceptance (Wong et al. 1994). Within the field of psychology, TMT researchers have demonstrated links between thoughts of death (Hirschberger and Shaham 2012) and the manipulation of death-related stimuli (Landau et al. 2004), to the concept of (im)permanence. Importantly, while the link between impermanence and death processing has not been researched in relation to psychedelics, the connection has not gone unnoticed. In their seminal book, ‘The Human Encounter with Death’, Grof and Halifax (1977) propose, based on psychedelic-assisted therapy, that psychedelics have the potential to induce a profound encounter with death and acceptance of human impermanence, leading to psychological transformation, including changes in perceptions of death. In sum, based on this wide body of literature, we propose that psychedelic-related changes in death processing are mediated by changes in the awareness and acceptance of impermanence as an organizing principle of human existence.

The last aim (Aim 3) of the project was to tie more firmly the candidate mechanisms-of-change (that is, the ones proving significant), to ayahuasca-related factors. For this purpose, exploratory analyses, within the ayahuasca group, examined whether ayahuasca usage habits (e.g., frequency, time since last use) and/or acute subjective experiences predicted any of the candidate mechanisms-of-change. For the latter, we were especially interested in the construct of ego dissolution which has been previously associated with numerous short and long-term effects, such as improvements in mental health and wellbeing, psychedelic-related therapeutic effect and nature-relatedness (for a review on this topic see Kałużna et al. 2022).

### Study aims and hypotheses

In summary, the current study aims are to (1) comprehensively examine group differences in death processing between ayahuasca veterans and non-users (control group), to (2) test candidate mediators of these group differences, and to (3) exploratorily examine whether ayahuasca-related factors are associated with them. In line with these aims, we hypothesized that (1) ayahuasca veterans would display a more ‘relaxed’ death processing system across six self-report and behavioral measures spanning death-related affective (anxiety, fear), cognitive (thought suppression), conceptual (an attitude of acceptance), and (avoidant) behaviors. Table 1 provides a summary of these measures, including their type (self-report/behavioral), the constructs they target, their domain, novelty of use in the psychedelic literature, and related hypotheses.

Additional measures, controlling for possible confounding variables, included a range of demographic variables and measures of psychopathology including depression (Beck et al. 1961) and anxiety (Spielberger et al. 1983). Additionally, as the study was conducted 1–2 years after the onset of the COVID-19 pandemic, we also measured COVID-related anxiety (Lee 2020) to account for any potential influence of pandemic-related concerns. For our second aim, we (2) tested ontological afterlife beliefs (Testoni et al. 2015), the big five personality dimensions of neuroticism and openness (John 1990), trait mindfulness (Baer et al. 2006), and impermanence awareness and acceptance (Fernández-Campos et al. 2021) as potential mediators of the group differences in death processing. For the third aim, we examined (3) whether ayahuasca usage habits including frequency, age of initial intake, time since last usage, time since strongest experience, and frequency of other psychedelic substances (LSD/psilocybin), and/or acute mystical experiences including typical and strongest ego dissolution experiences (Nour et al. 2016), predicted the (significant) mediators within the ayahuasca veterans group.

## Methods and materials

### Participants

Fifty-four experienced ayahuasca users and 53 matched non-users (the control group) were recruited via social media and personal connections. The groups were matched based on demographics, including gender, age and education. A power analysis (G*Power 3.1) verified that the present sample size was sufficient for detecting between-subject effects (*n* = 2 × 49 has power = 0.90 for a medium effect size *f* = 0.6 effect size at *α* = 0.05). To increase the likelihood of detecting group difference, participants with substantial ayahuasca experience were recruited. Inclusion criteria were: willingness to sign the informed consent, no current use of psychoactive medications (antidepressants, mood stabilizers, anxiolytics, and antipsychotics), no current use of drugs of abuse (e.g. cocaine), cannabis intake < twice a month, no neurological or active psychiatric illnesses (e.g., epilepsy, depression), and no loss of a first degree relative (spouse, parent, child, or sibling) within the last 12 months. In addition, specific inclusion criteria for the ayahuasca group were: multiple ayahuasca usage (≥ 9 times), considering ayahuasca as their primary psychedelic substance-of-use (their “medicine” of choice using the participants’ jargon), and no use of ayahuasca or other psychedelics in 28 days preceding the assessment to avoid afterglow effects (Evens et al. 2023). For the control group, no previous lifetime consumption of any psychedelic substances was required. Magnetoencephalography-compatibility inclusion factors including no history of head injury with loss of consciousness, not pregnant or lactating, no claustrophobia, and no metal implants. The study was approved by the Institutional Review Board of the Education Faculty, the University of Haifa, Israel. Overall, participants spent around 4 h at home (online) and in the lab completing the questionnaires and tasks (including MEG measurements) and were compensated for their time by a sum equivalent to 100 Euros.

### Measures

#### Demographics

Demographic items included age, gender, marital status, income, education, religion, and number of children.

#### Ayahuasca-related factors (ayahuasca group only)

*Ayahuasca consumption habits* included various lifetime ayahuasca-intake related factors associated to pharmacology and ayahuasca usage including frequency of use, age of first consumption, time (in months) since the last intake, and time since the strongest experience with ayahuasca. We also collected data regarding the lifetime usage of other psychedelics (psilocybin, LSD, mescaline).

*Acute subjective experiences* occurring during ayahuasca ceremonies were retrospectively measured via the Ego Dissolution Inventory (EDI) (Nour et al. 2016). The EDI is an 8-item self-report measure designed to capture past ego dissolution experiences. Each item in the inventory is rated using a visual analog scale format (0–100), where zero represents "No, not more than usual," and 100 represents "Yes, entirely or completely." Following the approach outlined in the original article by Nour et al. (2016), we used the EDI to assess both the "most intense" as well as “typical” past ego dissolution experience. The EDI was translated as described in the Procedure section. The resulting Cronbach’s alpha was 0.93, matching the one reported in the original EDI English study (Nour et al. 2016). 

#### Psychopathology measures

*Depression* was measured via the Beck Depression Inventory (BDI, Beck et al. 1961). The BDI is a validated tool commonly used in both clinical and research settings to assess the severity of depression and monitor changes in depressive symptoms over time. The measure consists of 21 items, with each item rated on a 0 to 3 scale, with higher scores indicating greater levels of depression (scores are summed to obtain a total score ranging from 0–63). Here we used a validated BDI Hebrew version (Lepkifker et al. 1988). The resulting Cronbach’s alpha coefficient was 0.86 as described in the literature (Beck et al. 1988).

*Anxiety* was measured with the State-Trait Anxiety Inventory-Trait measure (STAI , Spielberger et al. 1983). The STAI-Trait is a widely used and validated tool for assessing general trait anxiety level over time in both clinical and research settings. It consists of 20 self-report items, each rated as a 1–4 Likert scale, with higher scores indicating higher trait levels of anxiety (scores are summed to obtain a total score ranging from 20–80). We used a Hebrew version of the STAI questionnaire. The resulting Cronbach's alpha coefficient was 0.91, well within the range of the original published scale (Spielberger et al. 1983).

Covid anxiety was measured with Coronavirus Anxiety Scale (CAS, Lee 2020) given the context of the study conducted during the COVID-19 period (March 2021 – June 2022). The CAS, a newly developed 5-item scale, stands as an efficient and valid tool for assessing Coronavirus Anxiety. The CAS was translated as described in the Procedure section. The resulting Cronbach’s alpha was 0.81.

#### Death processing

In the present study, the term ‘death processing’ is used as an overarching construct denoting behavior, attitudes, as well as affective and cognitive processes associated with the theme of death. Specifically, the following measures were employed (see Table 1 for a summary):

*Death anxiety* was gauged via the Death Anxiety Scale (DAS, Templer, 1970). The DAS is the most widely used and validated tool applied in both clinical and research settings for assessing death anxiety levels and its impact on individuals' functioning. We employed the Likert scale version (Abdel-Khalek and Neimeyer 2017; Hayes and Gelso 1993) which consists of 15 items, each rated on a range from 1 (strongly disagree) to 5 (strongly agree). Total score is calculated by averaging the scores for all items, with higher scores indicating a greater level of explicit death anxiety. The DAS was translated as described in the Procedure section. The resulting Cronbach's alpha coefficient was 0.85, high and in the range of other studies using the translations of the scale (Abdel-Khalek and Neimeyer 2017).

*Death acceptance* was gauged via The Life Attitude Profile revised death acceptance sub-scale (LAP-RD, Reker 1981). LAP-RD is a reliable and valid 8-item measure of death acceptance. Each item on the LAP-RD is rated on a 7-point scale, ranging from 1 (“strongly disagree”), to 7 (“strongly agree”), with higher scores on this measure indicating greater explicit death acceptance. Previous studies demonstrate increased scores in LAP-RD after the use of psychedelics (Griffiths et al. 2016, 2018). The LAP-RD was translated as described in the Procedure section. The resulting Cronbach's alpha coefficient was 0.81, in the range of other studies using the translations of the scale (Mehnert and Koch 2008).

*Death behaviors avoidance* was gauged via the Death Behaviors Questionnaire (DBQ, Menzies 2020). DBQ is a 23-item questionnaire assessing how often several death-related situations are avoided (e.g., “visit a cemetery”, “think about yourself dying”, “read a novel with a character who is dying”). Each item is rated on a 5-point scale, ranging from 1 (“never avoid”), to 5 (“always avoid”), with high scores on the measure indicating greater death avoidant behavior. The DBQ was translated as described in the Procedure section. The resulting Cronbach's alpha coefficient was 0.91.

*Fear of death* was gauged via the Fear of Personal Death Scale (FPDS, Florian & Kravetz 1983). The FPDS is a widely used self-report scale measuring fear of death. It consists of 31 questions rated on a 7-point Likert scale, where respondents express their level of fear by responding to statements such as 'I am afraid of death because…'". In the present study, a modified version of the FPDS (Dor-Ziderman et al. 2019) was used, where the 31 original items were presented as a forced-choice yes–no computerized questionnaire. This presentation method allowed deriving an explicit and implicit measure relating to fear of death. The explicit measure was computed as the percentage of 'yes' responses (FPDS_P), with higher scores indicating a greater explicit fear of death. The implicit measure was computed by averaging the reaction times (RTs) associated with these responses and normalizing them by subtracting from them the latencies of a control (equally-long) 31-items fear of dental pain questionnaire (FPDS_RT). Following Dor-Ziderman et al. (2019), longer latencies for death-related items were interpreted as indicating increased emotional load. These results were replicated in the present study, where similar statistical differences were found (*t*(95) = 2.483, *p* = 0.01, *d* = 0.253), verifying that death-related items (mean = 4448.53 ms, *SD* = 1434.35, *SE* = 146.39) incurred longer RTs than dental-pain items (mean = 4201.88 ms, *SD* = 1180.11, *SE* = 120.44).

*Death-thought accessibility* refers to the 'level of activation of death thoughts', as theorized and experimentally demonstrated in TMT studies (see Hayes et al. 2010 for a review). According to TMT, thoughts of death are barred from entering conscious awareness and redirected away from focal attention when they arise (Hayes et al. 2010). Death-thought accessibility is measured using the Death Thought Accessibility (DTA) task, an implicit behavioral task word completion task. The task includes 20-word fragments, 10 of which can be completed with either a neutral or death-related word. For example, the fragment COFF_ _ could be completed as COFFEE (a neutral word) or COFFIN (a death-related word). Higher DTA scores indicate a greater number of words related to death being completed, reflecting increased death thought accessibility. It is important to note that while most TMT studies manipulate threats or conscious thoughts of death to examine their effects on DTA, other studies investigated individual differences of baseline DTA level as a predictor variable (Hayes et al. 2010), as is the current research approach.

#### Candidate mediation variables

*Personality* was assessed using the Big Five Inventory (BFI, John 1990), a commonly used personality assessment instrument that measures the five major personality traits, including openness (to experience), conscientiousness, extraversion, agreeableness, and neuroticism. The inventory consists of 44 items rated on a 5-point Likert scale (ranging from 1—strongly disagree to 5—strongly agree). Scores for each trait are calculated by averaging scores for the relevant items, with higher scores indicating higher levels of the trait. In the present study, we focused on openness and neuroticism, as these are the primary personality traits empirically shown to be related to psychedelic-induced changes. Specifically, psychedelic use is generally associated with an increase in openness (Erritzoe et al. 2018; MacLean et al. 2011; Madsen et al. 2020; Netzband et al. 2020) and a decrease in neuroticism (Erritzoe et al. 2018; Netzband et al. 2020; Weiss et al. 2021). We used a well-established Hebrew version of the BFI (Etzion and Laski 1998). The resulting Cronbach’s alpha coefficients for the BFI sub-scales were 0.81 for neuroticism and 0.79 for openness, which are within the range of other language translations (Alansari 2016).

*Ontological death representation.* Ontological representations of death as annihilation versus passage/continuation were measured via the Testoni Death Representation Scale (TDRS, Testoni et al. 2015). The TDRS is a 6-item self-report tool that assesses how individuals perceive death from an ontological perspective (e.g. “After I die, I will not exist anymore, so I will not experience anything”). Each item is rated on a 5-point scale, ranging from 1 (strongly disagree) to 5 (strongly agree). Lower TDRS scores indicate a perception of death as a passage/continuation, while higher scores signify a view of death as annihilation. The TDRS was translated as described in the Procedure section. The resulting Cronbach's alpha coefficient was 0.909.

*Impermanence *was gauged via the Impermanence Awareness and Acceptance Scale (IMAAS Fernández-Campos et al. 2021). The IMAAS scale includes 13 questions on a 7-point Likert scale, ranging from 1 (strongly disagree) to 7 (strongly agree). It consists of two factors: impermanence awareness, reflecting the cognizance that all phenomena are transient (IMAAS_AW) (e.g., " I know that aspects of myself will change with time "), and impermanence acceptance (e.g., "The idea that nothing in life lasts forever frightens me"), representing an attitude of openness towards the transient nature of all phenomena (IMAAS_ACC). Higher IMAAS_AW and IMAAS_ACC scores reflect greater impermanence awareness and acceptance, respectively.

*Mindfulness* was gauged via the Five Facets Mindfulness Questionnaire-Short Form (FFMQ) (Baer et al. 2006). The FFMQ is a 24-item validated self-report questionnaire that measures five mindfulness facets (observing, describing, acting with awareness, non-judging of inner experience, and non-reactivity to inner experience). Items in this questionnaire are scored on a 5-point Likert scale, ranging from 1 (never or very rarely true) to 5 (very often or always true). Higher scores indicate higher levels of mindfulness, and in the present study we used the total score as the dependent measurement of mindfulness abilities. The FFMQ was translated as described in the Procedure section. The resulting Cronbach's alpha coefficient was 0.79, which are within the range of translations from other language (Lilja et al. 2011).

### Procedure

*Questionnaires* were completed online via a web link to an anonymous survey using the Qualtrics survey tool (Qualtrics, Provo, UT). Questionnaires lacking validated Hebrew translations underwent translation using the back-translation method (Tyupa 2011) to ensure accuracy and cultural appropriateness for Hebrew speakers. This process involved one translator translating each questionnaire from English to Hebrew, followed by another independent translator translating them back from Hebrew to English. A third independent translator, who is a professional translator and editor, then assessed the translations to identify and rectify any discrepancies or errors.

*Behavioral measures* (FPDS and DTA) were administered at the Electromagnetic Brain Imaging Unit at Bar-Ilan University, Israel. The DTA task was administered using a paper sheet, and the FPDS task was administered while MEG was measured. It was presented on a screen using E-prime 3.0, with participant lying supine pressing a control box for collecting their responses.

*Missing data.* Most of the measures were completed by all of the participants (N = 107). Only 100 participants completed the DTA due to technical issues. In addition, the FPDS was completed by 98 participants: eight participants did not complete the task due to MEG-incompatibility factors, and one participant had to discontinue the task as the investigator needed to leave the lab for personal reasons. No statistically significant difference were observed between the ayahuasca users and non-users in terms of missing data for the DTA or FPDS measures (*χ*^*2*^ test, *p* > 0.05).

### Statistical analyses

Data analysis was conducted using SPSS version 23.0 (IBM Corp., Armonk, NY, USA) and jamovi software (version 2.3.1, The jamovi project). The internal consistency of the self-report scales was assessed using Cronbach's alpha coefficient (Cronbach 1951). Between-group comparisons (e.g. ayahuasca veterans vs. non-users controls) were conducted using two-tailed independent t-tests for continuous variables and and χ^2^ tests for categorical data.The assumption of homogeneity of variances was assessed prior to conducting the t-tests using Levene's test. When this assumption was violated (p < 0.05), Welch’s t-test was applied to account for the heterogeneity of variances. General linear models were used for comparisons involving more than two levels of factors, such as group and gender, followed by *t*-test post hoc analyses for further investigation. Pearson's (*r*) correlation coefficients were used for exploring correlations between measures. The significance threshold was set at 0.05. Mediation analyses were conducted separately for each possible mediator variable using the PROCESS macro for SPSS (Hayes 2013). Indirect effects were considered statistically significant if the confidence interval did not include zero. Multiple comparisons corrections were applied to our primary analyses (group differences in death processing and the candidate mediators underlying these differences) using the Holm-Bonferroni method for the *t*-tests, and by constraining the confidence interval to 99% in the mediation analyses. No corrections were applied to the exploratory analyses (examining whether ayahuasca usage habits or subjective experiences predict significant mediators). Effect size measures for group comparisons were computed using Cohen's d (*d*). Effect sizes for mediation models were quantified as the ratio of the indirect effect to the total effect, following the approach by Gaume et al. (2021).

## Results

The study findings are reported in compliance with the Strengthening the Reporting of Observational Studies in Epidemiology (STROBE) guidelines (Von Elm et al. 2007) for cross-sectional studies (checklist can be downloaded at https://www.strobe-statement.org/).

### Participants characteristics

Table 2 provides a summary of the study sample's characteristics, including demographic variables and psychopathology measures, including an assessment of group differences across these variables. The results showed no statistically significant differences between the groups in terms of psychopathology measures, Covid anxiety, past mental health diagnosis (i.e. depression/anxiety) and demographics, including gender, age, education, family status, and number of children. However, there was a difference between the groups in religion. Specifically, while 15.1% of participants in the non-users group identified themselves as ‘religious’, none of the participants in the ayahuasca group defined themselves as ‘religious’. On the other hand, in the ayahuasca group, 10.4% identified themselves as 'other', while in the non-users group, none of the participants identified themselves as 'other'. See the discussion section for more on this.

**Table 2:** Summary of study sample demographic and psychopathology characteristics. The table summarizes the characteristics (means, SD and the statistics) of the study sample, including demographic variables, and psychopathology, as a function of group (ayahuasca veterans or controls). Bold formatting indicates significant p values. *p* < 0.01

**Variable**	ayahuasca n = 54		controls n = 53	Total n = 107	Statistics
**Demographics**					
Age		38.5 ± 8.6	36.5 ± 7.7	37.5 ± 8.2	*t* = 1.27, n.s
Gender	Male Female	35 (32.7%) 19 (17.8%)	29 (27.1%) 24 (22.4%)	64 (59.8%) 43 (40.2%)	*χ*^2^ = 1.13, n.s
Education	High School or equivalent	10 (9.3%)	5 (4.7%)	15 (14%)	*χ*^*2*^ = 5.5, n.s
	College Diploma or certification studies	34 (31.8%)	28 (26.2%)	62 (57.9%)	
	Master’s Degree and above	10 (9.3%)	20 (18.7%)	30 (28.0%)	
Family status	Unmarried	25 (23.4%)	11 (19.6%)	46 (43.0%)	*χ*^*2*^ = 1.14, n.s
	Married	19 (17.8%)	24 (22.4%)	43 (40.2%)	
	Divorced	10 (9.3%)	8 (7.5%)	18 (16.8%)	
Income	Below average	12 (11.3%)	16 (15.1%)	28 (26.4%)	
	Average	23 (21.7%)	21 (19.8%)	44 (41.5%)	*χ*^*2*^ = 1.10, n.s
	Above average	19 (17.9%)	15 (14.2%)	34 (32.1%)	
Religion	Secular	34 (32.1%)	30 (28.3%)	64 (60.4%)	***χ***^***2***^ = 27.8*
	Traditional	9 (8.5%)	6 (5.7%)	15 (14.2%)	
	Religious	0 (0.0%)	16 (15.1%)	16 (15.1%)	
	Other	11 (10.4%)	0 (0.0%)	11 (10.4%)	
**Psychopathology**					
Psychiatric diagnosis		3 (5.6%)	2 (3.8%)	5 (4.7%)	*χ*^*2*^ = 1.91, n.s
Depression (BDI)		4.0 ± 4.14	4.70 ± 5.03	4.36 ± 4.59	*t* = 0.79, n.s
Trait anxiety (STAI)		36.7 ± 8.74	38.1 ± 9.32	37.4 ± 9.01	*t* = 0.79, n.s

**Table 3:** Summarizes the groups death processing variables and candidate mediators. Means, standard deviations (SD), and between-group (ayahuasca/controls) statistics are presented, including t-values, uncorrected *p*-values, corrected *p*-values (Holm-Bonferroni) within parentheses, Cohen’s *d* effect sizes, and 95% confidence intervals (CI) within brackets. Bold formatting indicates significant *p*-values

**Variable**	ayahuasca n = 54		control n = 53	Statistics (*t*, *p*, corrected *p*, *d*, 95% CI)
**Death processing variables**				
Death anxiety (DAS)		2.6±0.6	2.9 ± 0.6	*t* = −2.8**, *p* = 0.006 (0.018)**, *d*=−0.54, [−0.5 −0.1]
Death avoidance (DBQ)		1.8 ± 0.5	2.3 ± 0.6	*t* = −4.2, ***p* < 0.001 (0.004)**, *d*=−0.82, [−0.7 −0.2]
Death acceptance (LAP-RD)		5.0 ± 0.8	4.1 ± 1.2	*t* = 4.2, ***p* < 0.001 (0.005)**, *d*=0.81, [0.4 1.2]
Fear of death (FPDS_P)		0.1 ± 0.1	0.4 ± 0.2	*t* = −5.9, ***p* < 0.001 (0.006)**, *d*=−1.20, [−0.3 −0.1]
Death accessibility (DTA)		0.9 ± 0.9	1.1 ± 1.1	*t* = 0.7, *p* > 0.462
Fear of Death (FPDS_RT)		−18.3 ± 1023	565 ± 1183	*t* = −2.60, *p* = **0.01 (0.022)**, *d*=−0.52, [−1023, −136]
**Candidate mediators**				
Ontological beliefs (TDRS)		2.4±0.7	3.7±1.0	*T*=−6.9, ***p*<0.001 (0.005)**, *d*=1.33, [−1.5 −0.8]
Impermanence (IMAAS)	Awareness	6.2±0.9	5.6±1.1	*T*=3.0, ***p*=0.003 (0.009)**, *d*=0.59, [0.2 0.9]
	Acceptance	4.7±1.1	3.7±1.1	*T*=4.7, ***p* < 0.001 (0.004)**, *d*=0.91, [0.5 1.4]
Personality (BFI)	Neuroticism	2.4±0.6	2.7±0.6	*T*=−1.1, ***p*=0.032 (0.032)**, *d*=−0.43, [−0.5 −0.0]
	Openness	4.0±0.4	3.4±0.5	*T*=6.5, ***p*<0.001 (0.007),** *d*=1.27, [0.4 0.8]
Mindfulness (FFMQ)		3.7±0.3	3.5±0.3	*T*=3.0, ***p* = 0.003 (0.006)**, *d* = 0.21, [0.0 0.3]

Supplementary Table 1 displays the ayahuasca veterans group’s usage habits including lifetime use of ayahuasca (see Supplementary Fig. 1 for the distribution plot) and other psychdelics, age of first ayahuasca consumption, time (in months) since last ayahuasca intake and since strongest ayahuasca experience. Importantly, the findings re-inforce the unique role of ayahuasca (relative to other psychedelic substances) for our sample. On average, the participants consumed ayahuasca 55.7 (± 82.1) times, 5.2 times more than psilocybin (mean = 10.7 ± 15.4, *t* = 4.32, *df* = 51, *p* < 0.001, *η2* = 0.59), 4.6 times more than mescaline (mean = 12 ± 14.9, *t* = 3.40, *p* = 0.002, *η2* = 0.66), and 5.6 times more than LSD (mean = 9.9 ± 16.6, *t* = 4.5, *p* < 0.001, *η2* = 0. 67). Additional control analyses were conducted to confirm that the total lifetime use of other (not ayahuasca) psychedelics was unrelated to our variables, while ayahuasca lifetime use was correlated (though not surviving type I error correction) with death anxiety and FPDS RT (see Supplementary Table 2). It is essential to emphasize that the non-users group participants were pre-selected based on the condition that they reported no previous use of any psychedelic substances.

### Group differences in death processing

**Fig. 1:**
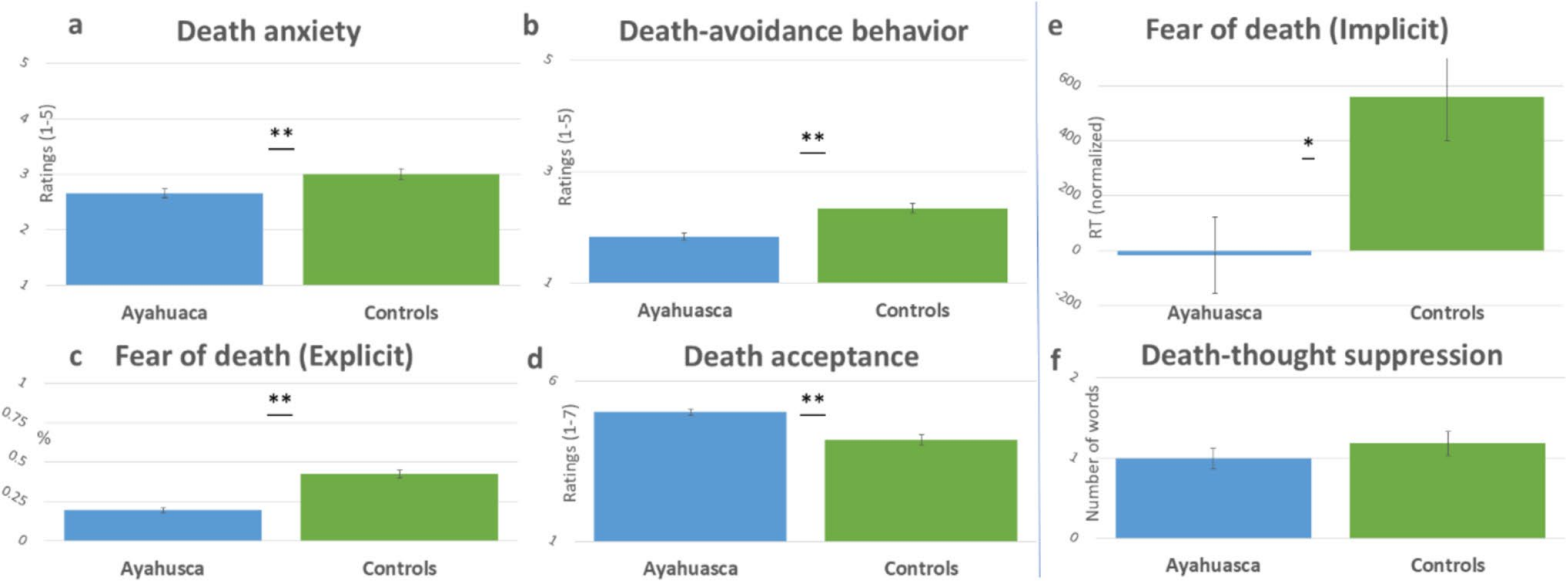
Group differences in death processing measures. Bar plots compare ayahuasca and non-users (x-axis) on various death processing measures (y-axis), including self-report measures of (**a**) death anxiety (DAS, mean), (**b**) death avoidant behavior (DBQ, mean), (**c**) explicit measure of fear of death (FPDS_P, % of ‘yes’ answers), (**d**) death acceptance (LAP-DA, mean); as well as behavioral measures of (**e**) implicit measure of fear of death (FPDS_RT, mean normalized RT), and (**f**) Death-thoughts suppression (DTA, number of words). Error bars represent the standard error of the mean. Statistical significance: uncorrected *p*-values ≤ 0.006 are denoted by **, and *p*-values = 0.01 are denoted by *. See Table 3 for exact values as well as Holm-Bonferroni corrected values (all remain significant)

The findings regarding the differences between the ayahuasca veterans and non-users control group on death processing factors largely aligned with our hypotheses. As detailed in Table 3 (group means ± SD, *t* statistics, corrected and uncorrected *p* values, and effect sizes), and illustrated in Fig. 1, ayahuasca users evidenced lower death anxiety, less death-avoidant behavior, less fear of death (both implicit and explicit), and higher death acceptance.

Based on consistent literature findings demonstrating higher DAS scores in women (Harding et al. 2005; Sawyer et al. 2021), we compared genders beyond the research groups (ayahuasca veterans and non-users) and identified a significant difference in DAS (*t* (105) = −2.07, *p* = 0.04, *d* = 0.408), with women scoring higher than males (mean for males = 2.73 ± 0.59, mean for females = 2.99 ± 0.70). No other gender differences were observed for other death processing measures (ps > 0.05). Following the significant DAS gender differences, we then tested for a group X gender interaction effect by computing a between-participant repeated-measures 2 × 2 ANOVA. The results (depicted in Supplementary Fig. 2), revealed a significant group X gender interaction (*F* (1,106) =8.08, *p* = 0.005, *η2* = 0.07). Post hoc tests revealed a significant gender difference for non-users (*t* (39.9) = −3.25, *p* = 0.02, *d* = −0.927, 95% *CI* [−0.8, −0.2], mean female DAS = 3.31 ± 0.709, mean male DAS = 2.76 ± 0.495), but not for the ayahuasca veterans group (*t* (52) = 0.712, *p* = n,s, 95% *CI* [−0.2, 0.4], mean female DAS = 2.58 ± 0.440, mean male DAS = 2.71 ± 0.676).

### Candidate mediators underlying the group differences

Candidate group-differences mediators included ontological afterlife beliefs, impermanence awareness and acceptance, mindfulness, and personality. As expected, all the candidate mediators evidenced significant group differences, such that ayahuasca veterans tended to believe more in death as passage (rather than annihilation), had higher values of both impermanence awareness and acceptance, were more open to experience and less neurotic, and scored higher on trait mindfulness (*p* < 0.05, Bonferroni-Holm corrected, see Table 3).

**Fig. 2:**
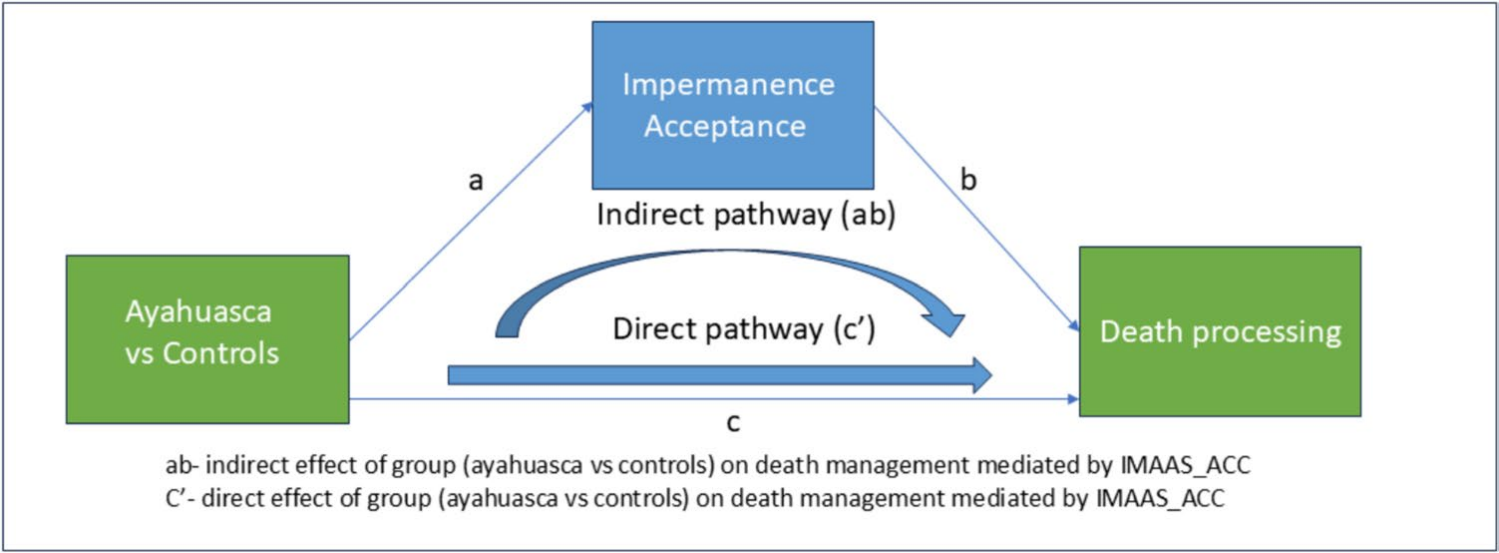
Mediation model depicting impermanence acceptance (IMAAS_ACC) mediation effects (path ab) on group (ayahuasca vs. controls) differences in death processing measures. Death processing measures include death anxiety (DAS), explicit fear of death (FPDS_P), death acceptance (LAP-RD), death avoidant behavior (DBQ), and implicit fear of death (FPDS_RT). See Table 4 for direct and indirect statistical mediation values and effect sizes

Mediation analyses (see Fig. 2) indicated that impermanence acceptance significantly mediated all of the death processing group differences, including implicit and explicit measure of fear of death, death anxiety, death acceptance, and death-avoidant behaviors. Table 4 supplies relevant statistical metrics, including direct ‘c’ and indirect effects ‘*ab*’, standard errors (*SE*), confidence intervals (*CI*), effect size (*ES*) for indirect path, as well as ‘*a*’ and ‘*b*’ paths. Given the significant gender differences in death anxiety observed in the previous section, an additional analysis controlling for gender effects was conducted. Results showed that impermanence acceptance still mediated (indirect effects) the effect of group (ayahuasca veterans vs. non-users) on death anxiety while controlling for gender (*ab* = 0.33, *SE* = 0.08, 99% *CI* 0.12 – 0.60). Mediation analyses for all the other candidate mediators did not yield significant results. That is, our results did not support impermanence awareness, afterlife beliefs, personality, and mindfulness, as mediators of the group differences of any of the death processing measures (see supplementary Table 3 for mediation metrics and statistics).

**Table 4:** Mediation models of death processing and impermanence acceptance (IMAAS_ACC) as mediator. Note: *SE* = Standard error; *CI* = Confidence interval, *ES* = Effect size (ratio of the indirect effect to the total effect). *CI* is estimated on 5,000 bootstrap samples. Abbreviations: LAP-RD (Life Attitudes Profile-Revised Death Acceptance Subscale), DBQ (Death Behaviors Questionnaire), DAS (Death Anxiety Scale), FPDS_RT (Fear of Personal Death Scale, RT delta Score), and FPDS_P (Fear of Personal Death Scale, Percentage Score)

	*a* path			*b* path			**Indirect effects (*ab*)**				Direct effects (*c*)		
Variables of interest	*B*	*SE*	[99% *CI*]	*B*	*SE*	[99% *CI*]	*Effect*	*SE*	[99% *CI*]	*ES*	*Effect*	*SE*	[99% *CI*]
LAP-RD	−1.0	0.21	−1.5 −0.45	0.39	0.08	0.17 0.62	−0.40	0.11	**−0.74 −0.16**	**0.45**	−0.47	0.20	−1.0 0.07
DBQ	−1.0	0.21	−1.5 −0.45	−0.16	0.05	−0.29 −0.03	0.16	0.06	**0.01 0.37**	**0.33**	0.33	0.12	0.01 0.66
FPDS_RT	−0.91	0.22	−1.5 −0.32	−316	96.6	−570.2 −62.1	288	122	**51.1 687.4**	**0.49**	291	230	−314 897
FPDS_P	−0.91	0.22	−1.5 −0.31	−0.00	0.01	00 −0.10	0.07	0.24	**0.02 0.14**	**0.30**	0.15	0.03	0.05 0.25
DAS	−1.0	0.21	−1.5 −0.45	−0.33	0.04	−0.45 −0.22	0.34	0.08	**0.14 0.58**	**1.0**	−0.00	0.10	−0.28 0.28

### Correlation analyses of ayahuasca-related factors with impermanence acceptance

Pearson correlation analyses between ayahuasca-related factors and impermanence acceptance (the sole significant mediator) indicated that only acute subjective experiences predicted degree of impermanence acceptance. Both ‘typical’ (EDI_T) and ‘strongest’ (EDI_S) ego dissolution measures positively correlated with impermanence acceptance (see Table 5). On the other hand, none of the ayahuasca-related intake habits (including lifetime use of ayahuasca, age of first ayahuasca consumption, time (in months) since the last ayahuasca intake, and time since the strongest ayahuasca experience) predicted impermanence acceptance (*p* > 0.05, see Table 5).

**Table 5:** Correlation between impermanence acceptance and ayahuasca-related factors. Abbreviations: EDI, Ego Dissolution Inventory; IMAAS_ACC,Impermanence and Acceptance Scale, Acceptance Subscale. Bold formatting indicates significant *p* values (< 0.05)

Ayahuasca-Related Factors		IMAAS_ACC
Acute subjective experiences	Strongest ego dissolution (EDI_S)	*r* = 0.26, ***p* = 0.04**
	Typical ego dissolution (EDI_T)	*r* = 0.32, ***p* = 0.01**
Consumption habits	Lifetime use of ayahuasca	*r* = 1.47, *p* = 0.28
	Age of first consumption	*r* = 0.05, *p* = 0.97
	Time since last intake	*r* = −0.03, *p* = 0.80
	Time since strongest experience	r = 0.10, p = 0.47

## Discussion

In the present cross-sectional study, we aimed to fill a gap in the literature and provide a comprehensive empirical assessment of altered death processing in a population of veteran ayahuasca users, to investigate putative underlying mechanisms-of-change, while linking them with ayahuasca-related acute subjective experiences. Overall, the findings validate our first hypothesis and make a solid case for a more ‘relaxed’ generalized death processing system in ayahuasca veterans. These long-term ayahuasca users displayed less explicit and implicit fear of death, less death-related anxiety, more death acceptance, and less death-avoidant behavior. Only the DTA measure, which targets the degree of death thoughts accessibility, did not differ between the groups, possibly due to a floor effect. The participants of both groups completed ambiguous word stems (which could generate both death-related and non-death-related words) as death-related words, on average, about once out of a possible ten. Future studies may benefit by using study designs that allow measuring DTA following a mortality salience manipulation (with or without a delay), as is typical in TMT studies (Hayes et al. 2010). Another finding, which we didn’t hypothesize, but which may be of interest to the literature regards an interaction between ayahuasca usage and gender on death anxiety (supplementary Fig. 2). Our findings on non-users are in line with the literature which reports higher death anxiety for women (Harding et al. 2005; Sawyer et al. 2021). However, in the ayahuasca group, no gender differences in death anxiety were observed. These novel results – though requiring replication – possibly indicate that ayahuasca may be especially beneficial for women in reducing their death anxiety.

While this study is the first cross-sectional investigation of death processing among ayahuasca users and non-users, its findings are consistent with the limited evidence gathered from online survey studies following ayahuasca usage which indicate less fear and anxiety of death (Griffiths et al. 2019; Sweeney et al. 2022). Likewise, the results are consistent with research on other psychedelics (e.g., LSD and psilocybin), which demonstrate a similar pattern: an increase in death acceptance and a decrease in death anxiety and fear among psychedelic users or following psychedelic experiences (Davis et al. 2020; Gasser et al. 2015; Griffiths et al. 2011, 2016, 2018, 2019; Moreton et al. 2023a; Sweeney et al. 2022; Yaden et al. 2017). However, the present findings go beyond previous studies, which assessed death processing using a singular self-report metric. By targeting affective, conceptual, cognitive, and behavioral aspects of death processing, and using both explicit and implicit measures, the current findings offer a comprehensive view of how the human psyche interacts with the theme of death, revealing a ‘relaxing’ of the death processing system over multiple levels of human cognition, affect, and behavior.

Given the inherent shortcomings of cross-sectional designs (Wang and Cheng 2020), measures were taken to control for confounding variables by balancing the samples over a large array of demographic factors including age, gender, family status, education, income, and religion. In our sample, only religion showed group differences. 15.1% of the non-users identified as religious, compared to none of the ayahuasca users. Conversely, 10.4% of the ayahuasca veterans group identified as 'other', compared to none in the non-users group. We are not overly concerned with this discrepancy as first, these differences concern a small portion of the sample. Second, it is likely that the ‘other’ option chosen by 10.4% of the ayahuasca participants relates to the ‘spiritual but not religious’ denomination (Kenneson 2015), as entheogenic spirituality falls under the New Age umbrella (Johnstad 2022). Possibly, the category 'other' reflects what Lee (2014) refer to as ‘non-nominal’ identities—nonreligious identifications that appeal to people who seek to reject categorization regarding (non)religiosity spectrum. Future studies should investigate the nature of these differences and other ethnoreligious difference, especially among ayahuasca users, due to the strong religious contextual influence of ayahuasca (Dupuis 2022). In particular, the unique settings of ayahuasca usage in Israel, which combine elements of New Age spirituality with Jewish religious traditions (Hartogsohn and Zadoff 2023; Mishor 2016), warrant further research.

An important contribution of the present study was running mediation analyses on several variables which were likely to account for group differences in death processing – either due to baseline population differences, or as a result of lifetime psychedelic use. That is, these analyses served a dual role, on the one hand, they allowed controlling for variables which may be characteristic of the groups beyond ayahuasca usage; and on the other hand, as these variables are also known to be impacted by psychedelics, mediation analyses allow examining their role as putative mechanisms-of-change. In line with relevant literature, the findings indeed show that ayahuasca veterans are more open to experiences (Barbosa et al. 2016), tend to more strongly view death as passage rather than annihilation (ontological beliefs, see next paragraph), and are more mindful as a trait (Sampedro et al. 2017). While the present design cannot determine whether these differences are indeed due to lifetime ayahuasca usage, they do not account for any of the group differences regarding the different death processing measures.

The results concerning ontological beliefs are especially interesting as they run contrary to a recent paper (Letheby 2024) which argues for it being the underlying mechanism-of-change accounting for the reduction in fear and anxiety of death following psychedelic usage. This theory, termed by Letheby (2024) the Metaphysical Belief Theory of psychedelic therapy, posits that the beneficial psychological effects of psychedelics on reducing fear and anxiety of death stem from an increase in non-physicalist beliefs. While, as reviewed in the introduction, there is a strong case to be made for such beliefs being impacted by psychedelics (Griffiths et al. 2011, 2018; Nayak et al. 2023; Timmermann et al. 2021), their link and causal relation to the theme of death is not well established. While presenting a thorough examination of the literature’s theoretical and anecdotal evidence, the only direct empirical evidence Letheby (2024) provides is a recent exploratory retrospective study (*n* = 155, including 39 ayahuasca users) demonstrating that reductions in death anxiety were weakly correlated with psychedelic-related changes towards panpsychism (the belief that consciousness is a fundamental and ubiquitous aspect of all matter) (Moreton et al. 2025). However, the same study also tested for similar correlations with 12 other belief items indexing an array of metaphysical beliefs (e.g., Ontological transcendentalism, Non-naturalism, etc.…, based on the questionnaire developed in Timmerman et al. 2021) – none of which were significant. We contend that more robust empirical evidence is necessary for a hypothesis with such important ramifications. As Letheby (2024) writes: “What are the options for a physicalist atheist who fear death and loves truth?”. Our results suggest that such individuals could benefit from psychedelic interventions for reducing fear and anxiety of death while maintaining their materialist world views. This information is important in light of recent ethical discussions on psychedelic induced “epistemic harm” (i.e. psychedelics may promote the adoption of false or delusional beliefs) (McGovern et al. 2024), and difficult questions regarding the ability to medically consent to psychedelic-induced belief changes (Jacobs 2023). While addressing these issues is beyond the scope of the current paper, our findings do establish that changed ontological beliefs are not necessary preconditions for changing one’s approach towards death.

Rather, our results make a case for acceptance of impermanence as a robust and specific mechanism-of-change underlying group differences in death processing. Impermanence acceptance robustly mediated the group differences on *all* the death processing measures (death anxiety, avoidant behavior, explicit and implicit fear of death, and death acceptance), while the other potential mediators (mentioned above), including impermanence awareness, did not mediate *any* of the death processing measure. While the concept of impermanence is central in Buddhist traditions, it is a profound cross-cultural concept that has its roots in ancient Western philosophy going back to Heraclitus and his doctrine of *panta rhei*, that ‘everything flows’ (Weisel 2010). Impermanence underscores the uncertainty and constant change inherent in existence, which may not be immediately available to human experience, but is built in current scientific frameworks (Prigogine 1997). These features are also compatible with current frameworks of brain function which view the brain as a ‘prediction machine’, whose sole purpose is the reduction of uncertainty by constantly updating (changing) its simulations of a hidden reality based on incoming sensory evidence (Clark 2013; Friston 2010; Hohwy 2013). This view, which is also influential in understanding the effects of psychedelics on brain function and ensuing long-term changes (Carhart-Harris and Friston 2019), aligns with the notion of impermanence as a basic feature underlying human cognition. Thus, impermanence acceptance, the deeply felt acceptance of change and uncertainty – including that of human life – can be viewed as an alignment of human conceptual understanding with the way things are, as well as with the way its cognitive apparatus functions.

**Fig. 3:**
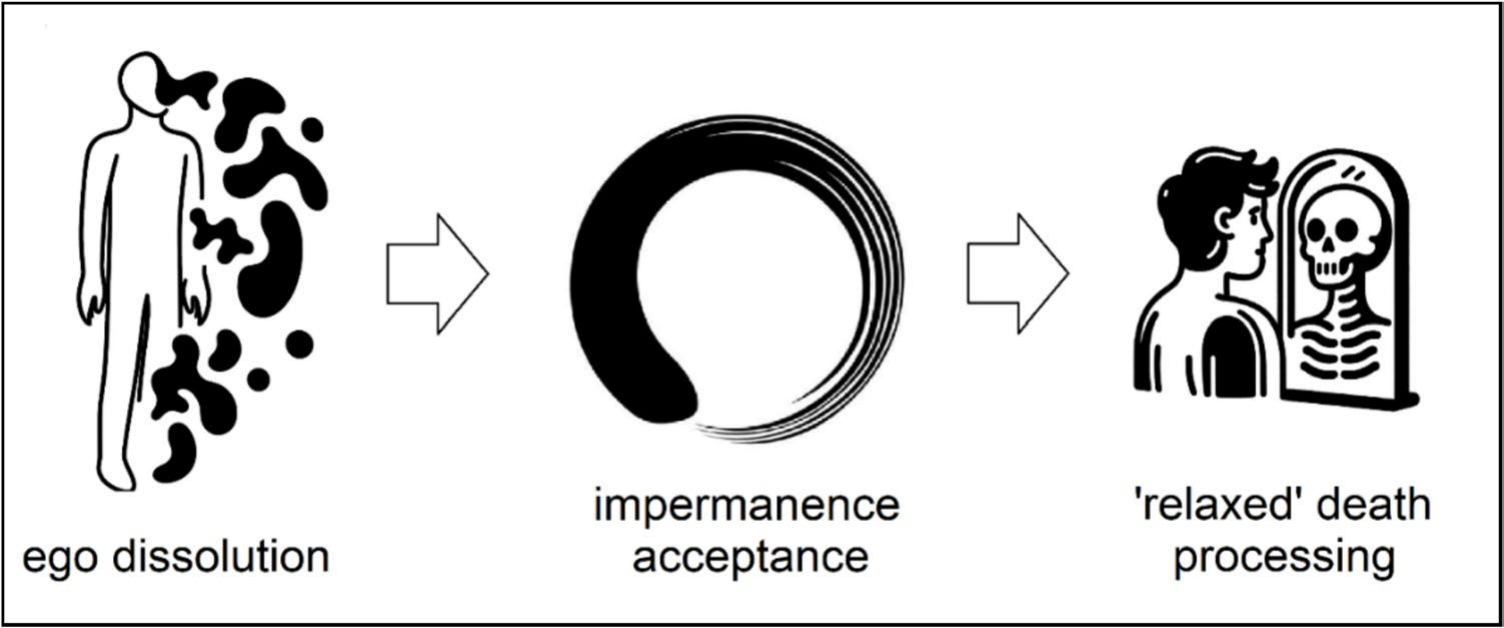
Explanatory model based on the study results for how ayahuasca, and psychedelics more generally, impact death processing through the cultivation of impermanence acceptance. Arrows indicate hypothesized causal effects. Images created using DALL·E

The reported association between degree of impermanence acceptance and strength of lifetime acute ego dissolution experiences among ayahuasca veterans provide initial evidence (though correlational) that levels of impermanence acceptance are modulated by ayahuasca-related factors, and more specifically, acute consciousness-altering experiences occurring during ceremonies. On the other hand, no associations were found between impermanence acceptance and measures related to pharmacology and ayahuasca use itself (e.g. lifetime use, time since last use, etc.). These findings align with other studies highlighting the role of acute subjective effects (Yaden and Griffiths 2021), and ego dissolution in particular, as causal in incurring the long-term effects of psychedelics in general (Kałużna et al. 2022), and ayahuasca in particular (Uthaug et al. 2018; Van Oorsouw et al. 2021). But why do such psychedelic mystical-type experiences induce an acceptance of impermanence? One possible answer is that under acute psychedelic conditions, the deepest layers of human brain temporarily become malleable, yielding radically different experiences of the sense of self, logical frameworks for understanding the world, and ordinary states of being (Carhart-Harris and Friston 2019). These extreme temporary experiences, which under acute psychedelic influence may seem to be irreversible, that there is 'no way back', reliably subside as the hallucinogenic molecules dissipate and lose their potency. This notion was beautifully expressed by one of our participants, whose quote at the beginning of the illustrates the reliable fading and waxing of even the most taken-for-granted facets of reality, which can be as frightening as they are awe-inspiring, may lead to an experiential understanding and acceptance of impermanence as inseparable from reality. A deeply felt acceptance that nothing in our experience, including life itself, is immune to change (see Fig. 3 for a schematic illustration of the hypothesized process leading to ‘relaxed’ death processing).

The present findings are relevant to the growing recognition and interest in psychedelics as a promising therapeutic option for individuals experiencing end-of-life existential distress (Reiche et al. 2018). However, a recent review of registered and ongoing trials on psychedelics for end-of-life care reported 25 trials, none of which included ayahuasca (Jing et al. 2023). Considering the present results, it may be timely to conduct clinical research with ayahuasca interventions for end-of-life populations. Such interventions can also incorporate contemplative components targeting impermanence, possibly in combination with meditation. Psychedelic interventions often incorporate mindfulness elements as an integral part of their design with good results (Griffiths et al. 2018; Smigielski et al. 2019a, b; Smigielski, Scheidegger, et al., 2019). Furthermore, neurophenomenological (Berkovich-Ohana et al. 2020) studies of mindfulness meditation practice show reliable neural (Dor-Ziderman et al. 2013, 2016; Trautwein et al. 2024) and phenomenological (Ataria et al. 2015; Nave et al. 2021) correlates of meditative self-dissolution, an experience which shares similarities with psychedelic-induced ego dissolution (Millière et al. 2018). Furthermore, a recent study found an inverse relation between the neural correlates of death denial (Dor-Ziderman et al. 2019) and the valence of meditative self-dissolution experiences (Dor-Ziderman‬ et al. 2025). Thus, future studies can explore the long-term outcomes of death processing and impermanence associated with psychedelics compared to meditation, as well as add to the literature examining their synergistic effects (Eleftheriou and Thomas 2021; Payne et al. 2021;), especially in relation to ego dissolution (Smigielski et al. 2019a, b). However, we are not aware of any psychedelic interventions which directly aim at targeting impermanence acceptance during the mindset and integration phases. In fact, as pointed out by Fernández-Campos et al., (2021), meditations focusing on impermanence are rarely included in contemplative trainings which tend to focus on mindfulness and compassion practices. In this regard, it is interesting to mention a recent randomized controlled trial which contrasted a 6-week mindfulness meditation to a matched Christian contemplative practice (Anālayo et al. 2022). Both interventions similarly reduced fear of death and dying, and both incorporated meditation/contemplation (respectively) modules directly focusing on impermanence.‬‬‬‬‬‬‬‬‬‬‬‬‬‬‬‬‬‬‬‬‬‬‬‬‬‬‬‬‬‬‬‬‬‬‬‬‬‬‬‬‬‬‬‬‬

Despite the promise of ayahuasca-based interventions, it is important to acknowledge that although both the literature and our current results generally show a more 'relaxed' approach to death among psychedelic users, some individuals may experience heightened fear and anxiety about death following psychedelic experiences (Griffiths et al. 2019; Moreton et al. 2025; Moreton et al. 2023a). This aligns with studies showing that a certain percentage of individuals undergoing psychedelic experiences develop worsened mental health (Bouso et al. 2022; Dos Santos et al. 2017; Marrocu et al. 2024). Thus, in applying these insights to future interventions, it will be crucial to identify risk predictors and employ pre-intervention screening. No less important, implementation of post-psychedelic psychological integration and support, fostering rapport and trust between the user and facilitator, will help mitigating adverse psychedelic-induced negative psychological outcomes (Johnson et al. 2008). It's also conceivable that specific integration protocols should be developed for those undergoing (difficult) death-related psychedelic experiences.

The current study has several limitations. Firstly, the study's cross-sectional design does not allow for the attribution of causality to any of the reported results. Thus, future studies should test the results using longitudinal designs. Secondly, the findings, though showing robust statistical effects, are based on a relatively small sample size and therefore at risk of type 2 error, particularly for self-reports measures. Thirdly, although our sample consists of ayahuasca veterans who used ayahuasca significantly more than other psychedelics and considered it their primary ‘medicine’, the potential influence of other psychedelics cannot be entirely ruled out. Despite our statistical controls, further research is needed to definitively rule out the impact of other psychedelics. This could be addressed in future studies by studying ayahuasca veterans with no history of other psychedelic use – though identifying such a population may be challenging. Fourthly, generalizing our findings to ayahuasca use in general, rather than to populations of long-term veteran users is challenging due to a potential (self-)selection bias. Our sample included experienced users with no active psychiatric disorders, representing a specific cohort which may not be representative of ayahuasca users in general. Long-term use implies having had positive experiences with the ayahuasca brew, while those with more negative experiences may have discontinued use, potentially excluding them from the study. Despite these limitations, the present study makes a significant and innovative contribution to our understanding of death processing and its mechanisms among ayahuasca and psychedelic users. Furthermore, we believe our results have more general implications for coping with existential distress. By identifying impermanence acceptance as an efficacious mechanism-of-action, future research and interventions—both psychedelic and non-psychedelic—can directly target this construct to manage death anxiety in clinical and non-clinical settings. Finally, these results are consistent with studies suggesting that the naturalistic use of ayahuasca may be associated with beneficial effects in certain populations (Domínguez-Clavé et al. 2019, 2022; Frecska et al. 2012; González et al. 2020; Jiménez-garrido et al. 2020; Kiraga et al. 2021; Kohek et al. 2022; Murphy-Beiner and Soar 2020; Ona et al. 2019; Perkins et al. 2022; Soler et al. 2018; Uthaug et al. 2018; Van Oorsouw et al. 2021). In this context, repeated ayahuasca use may aid the human psyche in coming to terms with its finitude and managing existential terror.

## Supplementary Information

Below is the link to the electronic supplementary material.

Supplementary file1 (DOCX 148 KB)

## Data Availability

Data will be made available upon request.
