# Supplementary material for: Embracing change: impermanence acceptance mediates differences in death processing between long-term ayahuasca users and non-users
Source: Psychopharmacology (Berl). 2025 Apr 23;242(10):2201–18. doi: 10.1007/s00213-025-06792-0 (PMC12449329; doi:10.1007/s00213-025-06792-0)
Supplement: Supplementary file 1 — Supplementary file1 (DOCX 148 KB) [file 213_2025_6792_MOESM1_ESM.docx]

**Supplementary Information**

Supplementary Table 1: Lifetime usage parameters of ayahuasca and other psychedelics

| **ayahuasca** | Lifetime use (usage frequency) | 55.8 ± 82.1 |
| --- | --- | --- |
|  | Age of first ayahuasca use | 31.7 ± 8.2 |
|  | Last use of ayahuasca (month) | 8.2 ± 5.2 |
|  | Time since most strong ayahuasca (month) | 33.8 ± 35.5 |
| **Other psychedelics** | Lifetime use of LSD | 9.9 ± 16.6, N = 43 |
|  | Lifetime use of mescaline | 12 ± 14.9, N = 26 |
|  | Lifetime use of psilocybin | 10.7 ± 15.4, N = 52 |

Supplementary Figure 1: Illustration of the distribution of ayahuasca usage within the sample.


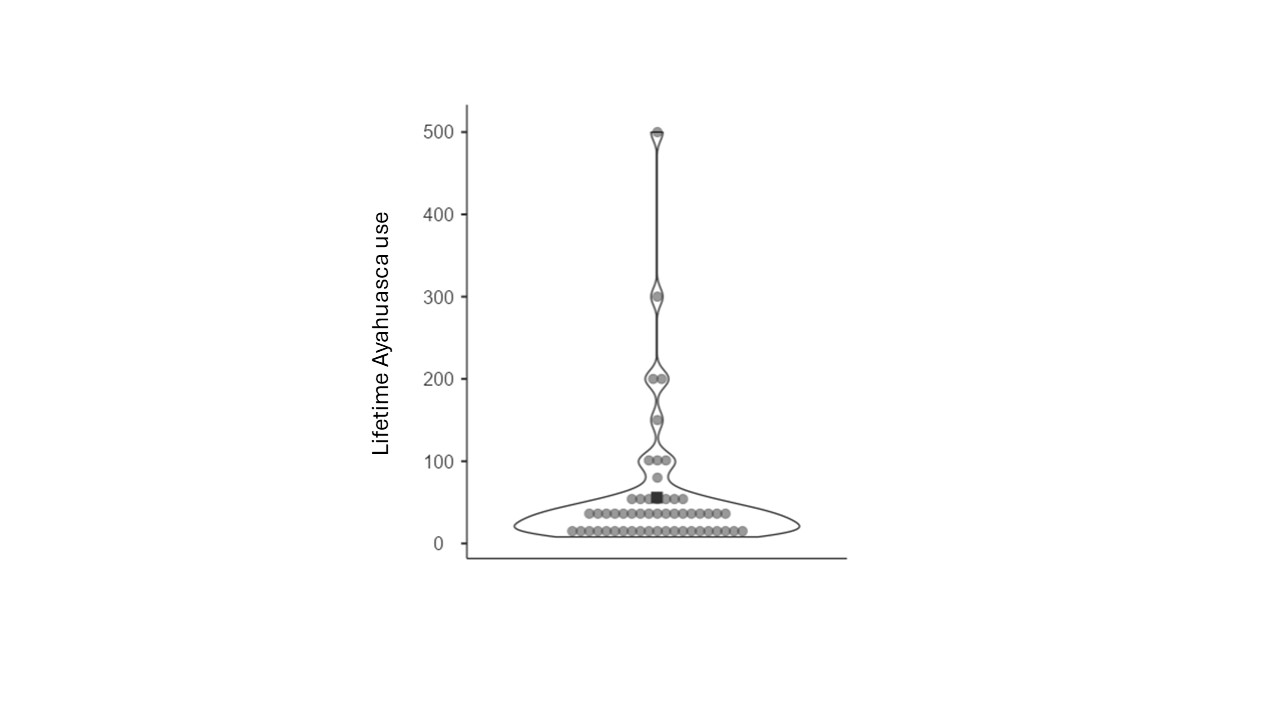


**Supplementary Figure 1.** The distribution of ayahuasca usage within the sample. The y-axis represents the number of lifetime ayahuasca uses, while the x-axis represents individual subjects depicted as circles. The square represents the mean.

Supplementary Figure 2: Death anxiety as a function of gender and group
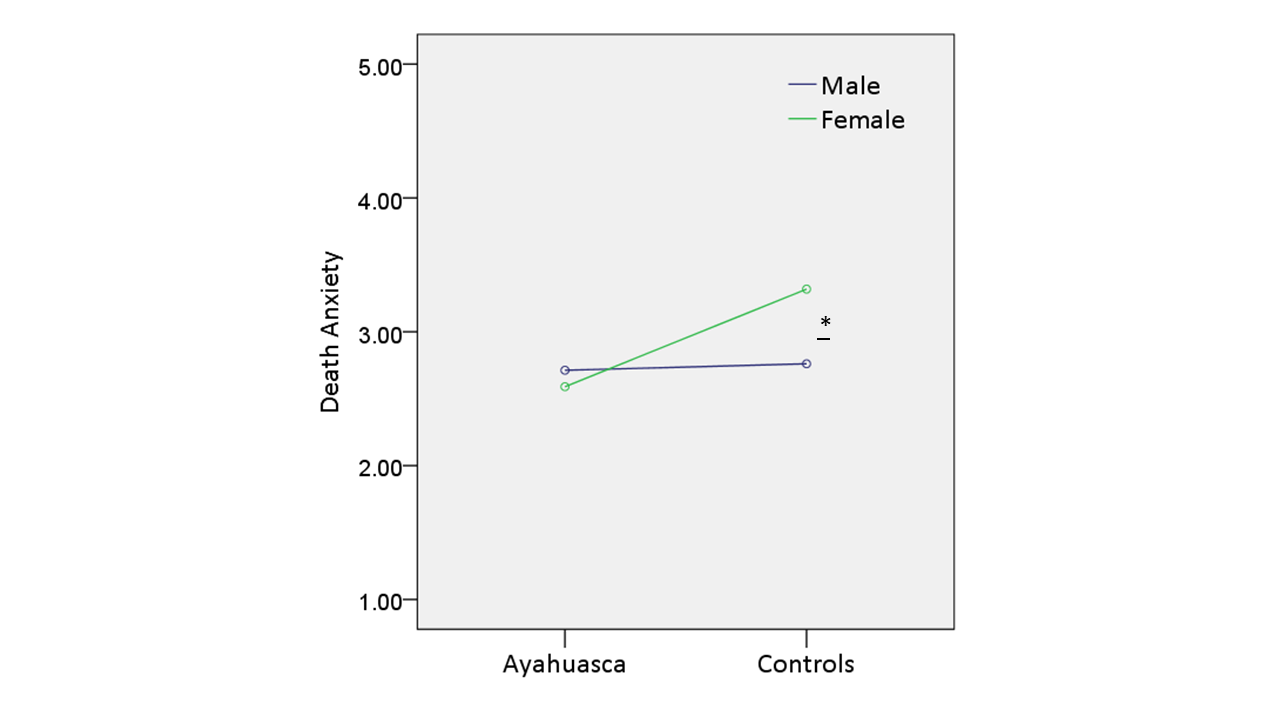


**Supplementary Figure 2.** Interaction between groups (ayahuasca and controls, X-axis) and gender (blue line for male, green line for female) on the Death Anxiety Scale (DAS (Templer, 1970), Y-axis). Statistical significance: *p*-values = 0.02 is denoted by *.

Supplementary Table 2: Correlation between Lifetime Ayahuasca and Lifetime Sum of Other Psychedelics with All Research Variables

| **Type** | **Variable** | **LT Ayahuasca (55.7 ± 82.1) (r, p)** | **LT Psychedelics (41.1 ± 48.6) (r, p)** |
| --- | --- | --- | --- |
| Death processing variables | Death anxiety (DAS) | **r = -0.284, p = 0.037** | r = -0.244, p = 0.274 |
|  | Death avoidance (DBQ) | r = -0.159, p = 0.249 | r = -0.216, p = 0.333 |
|  | Death acceptance (LAP-RD) | r = 0.139, p = 0.315 | r = 0.131, p = 0.562 |
|  | Fear of death (FPDS_P) | r = -0.107, p = 0.463 | r = -0.098, p = 0.672 |
|  | Death accessibility (DTA) | r = -0.021, p = 0.886 | r = -0.123, p = 0.585 |
|  | Fear of Death (FPDS_RT) | **r = -0.360, p = 0.011** | r = -0.094, p = 0.686 |
| Candidate mediators | Ontological beliefs (TDRS) | r = -0.111, p = 0.425 | r = -0.190, p = 0.397 |
|  | Impermanence (IMAAS) | | |
|  | Awareness | r = 0.147, p = 0.288 | r = 0.249, p = 0.264 |
|  | Acceptance | r = 0.007, p = 0.961 | r = 0.066, p = 0.770 |
|  | Personality (BFI) | | |
|  | Neuroticism | r = -0.131, p = 0.347 | r = -0.284, p = 0.201 |
|  | Openness | r = 0.205, p = 0.137 | r = 0.034, p = 0.881 |
|  | Mindfulness (FFMQ) | r = -0.048, p = 0.733 | r = 0.354, p = 0.106 |
| Acute subjective experiences | Strongest ego dissolution (EDI_S) | r = 0.065, p = 0.641 | r = -0.021, p = 0.926 |
|  | Typical ego dissolution (EDI_T) | r = 0.108, p = 0.438 | r = 0.125, p = 0.579 |

Supplementary Table 2. Correlation between Lifetime Ayahuasca and Lifetime Sum of Other Psychedelics with All Research Variables. Abbreviations: LAP-RD (Life Attitudes Profile-Revised Death Acceptance Subscale), DBQ (Death Beliefs Questionnaire), DAS (Death Anxiety Scale), FPDS_D (Fear of Personal Death Scale, Delta Score), FPDS_P (Fear of Personal Death Scale, Percentage Score), DTA (Death Thought Accessibility), TDRS (Transcendence of Death-Related Self), IMAAS_ACC (Impermanence and Acceptance Scale, Acceptance Subscale), IMAAS_AWARENESS (Impermanence and Acceptance Scale, Awareness Subscale), BFI (Big Five Inventory), FFMQ (Five Facet Mindfulness Questionnaire), EDI (Ego Dissolution Inventory). Bold formatting indicates significant p-values (<.05).

Supplementary Table 3: Mediation models of group differences in death processing of candidate mediators other than impermanence acceptance

| Variables of interest | | Indirect effects | | |
| --- | --- | --- | --- | --- |
| Impermanence awareness (IMAAS_AW) |  | *Effect* | *SE* | [95% *CI*]* |
|  | LAP-RD | .0357 | .0664 | -.2195 .1838 |
|  | DBQ | .0689 | .0476 | -.0088 .2396 |
|  | FPDS_D | -100.9360 | 67.2648 | -305.4484 102.4071 |
|  | FPDS_P | -.0180 | .0203 | -.0794 .0431 |
|  | DAS | -.0180 | .0203 | -.0794 .0431 |
| Ontological beliefs (TDRS) | LAP-RD | -.1453 | .1513 | -.5894 .2572 |
|  | DBQ | -.0049 | .0894 | -.2661 .2236 |
|  | FPDS_D | -127.7018 | 139.8506 | -563.2728 225.5430 |
|  | FPDS_P | .0204 | 0334 | -.0749 .1119 |
|  | DAS | -.1705 | .0859 | -.4251 .0298 |
| BFI Neuroticism | LAP-RD | -.0792 | .0652 | -.3048 .0274 |
|  | DBQ | .0261 | .0297 | -.0452      .1276 |
|  | FPDS_D | 100.5156 | 61.0976 | -32.7250 299.3505 |
|  | FPDS_P | -.0108 | .0130 | -.0614 .0107 |
|  | DAS | .1120 | .0582 | -.0222 .2962 |
| BFI Openness | LAP-RD | .1533 | .1465 | -.2171 .5675 |
|  | DBQ | -.0329 | .0274 | -.1086 .0407 |
|  | FPDS_D | 63.8791 | 133.9408 | -307.5799 430.0007 |
|  | FPDS_P | -.0329 | .0282 | -.1126 .0453 |
|  | DAS | -.0199 | .0927 | -.2714 .2437 |
| Mindfulness (FFMQ) | LAP-RD | -.0974 | .0735 | -.3460 .0638 |
|  | DBQ | .0830 | .0409 | -.0020 .2173 |
|  | FPDS_D | 55.8151 | 62.7440 | -87.0538 275.2259 |
|  | FPDS_P | .0137 | .0105 | -.0095 .0463 |
|  | DAS | .0860 | .0474 | -.0042 .2464 |

**Supplementary Table 3.** Mediation models of death processing and mediators. Note: *SE* = Standard error; *CI* = Confidence interval, * Estimated on 5,000 bootstrap samples. Abbreviations: LAP-RD (Life Attitudes Profile-Revised Death Acceptance Subscale (Reker, 1992)), DBQ (Death Beliefs Questionnaire(Menzies, 2019)), DAS (Death Anxiety Scale), FPDS_D (Fear of Personal Death Scale, Delta Score), and FPDS_P (Fear of Personal Death Scale, Percentage Score)(Dor-Ziderman et al., 2019; Florian and Kravetz, 1983).

ראש הטופס
